# Innovations in glaucoma treatment: development and testing of a novel self-adjustable implant for effective pressure regulation

**DOI:** 10.1038/s41378-026-01400-3

**Published:** 2026-07-27

**Authors:** Soroush Rafiei, Julien Maxime Gerber, Stéphane Bigler, Adan Villamarin, Constantinos Stergiopulos, Nikolaos Stergiopulos

**Affiliations:** 1 Laboratory of Hemodynamics and Cardiovascular Technology (LHTC), Swiss Federal Institute of Technology (EPFL), Lausanne, Switzerland; 2 Rheon Medical SA, EPFL Innovation Park, Building B, Lausanne, Switzerland

**Keywords:** Engineering, Physics

## Abstract

Glaucoma drainage devices (GDDs) are widely used to lower intraocular pressure (IOP) and slow disease progression; however, existing designs often lack intrinsic protection against early postoperative hypotony, show limited adaptability to evolving distal outflow resistance during bleb maturation, and rely on bulky or complex components that constrain implantation and MRI compatibility. Here, we present a compact, fully passive, self-adjustable glaucoma implant designed to maintain IOP within the physiological range by dynamically modulating its hydraulic resistance without external control or postoperative adjustment. The device consists of a circular microvalve incorporating a thin, pre-stressed compliant membrane that responds to pressure conditions at the inlet and outlet. Valve performance was investigated using fully coupled fluid–structure interaction (FSI) simulations and parametric analyses, followed by fabrication and in vitro characterization, and ex vivo assessment of surgical feasibility in enucleated porcine eyes. The results demonstrate effective pressure regulation across physiologically relevant conditions, with mitigation of early hypotony and attenuation of pressure elevation under increasing downstream resistance. Ex vivo implantation confirmed ease of placement, appropriate anatomical fit, and compatibility with standard surgical workflows. Owing to its compact form factor, passive operation, and simplified, cost-effective design, the proposed implant addresses key limitations of current GDDs and shows strong potential for clinical translation.

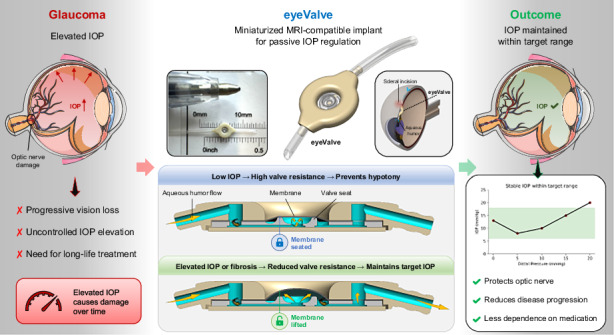

## Introduction

Glaucoma is the leading cause of irreversible blindness worldwide and affects more than 70 million people^1^. With aging populations and increasing life expectancy, its prevalence is projected to rise from approximately 76 million in 2020 to more than 110 million by 2040^2,3^. The hallmark modifiable risk factor in glaucoma is elevated intraocular pressure (IOP), which results from an imbalance between the production and drainage of aqueous humor (AH), the clear fluid that continuously fills the anterior chamber (AC) to maintain ocular shape and support optical function. AH is produced by the ciliary body and normally exits the AC mainly through drainage pathways located at the iridocorneal angle. In glaucoma, increased resistance within these outflow pathways impairs AH clearance, leading to fluid accumulation and chronically elevated IOP. Pressure lowering remains the central objective of glaucoma management to prevent progressive optic nerve damage and vision loss, and first-line treatments typically include topical medications and laser procedures^4,5^. However, when these approaches fail to maintain IOP within the target range, surgical interventions are required to create an alternative drainage route from the AC and thereby reduce IOP^6^, including implantation of glaucoma drainage devices (GDDs).

GDD surgery, often referred to as tube-shunt surgery, remains a mainstay of surgical glaucoma care and is frequently favored by glaucoma specialists. Large population-level datasets across multiple health systems further show that its use has continued to increase, particularly in complex or higher-risk eyes and in situations where trabeculectomy is expected to fail^7–10^. Modern GDDs build on the tube-and-plate concept introduced by Molteno, which diverts AH from the AC to the subconjunctival space to form a drainage reservoir around an end plate^11^. The Baerveldt implant, introduced in 1990, refined this architecture with silicone tubing and a flexible silicone plate incorporating barium for radiopacity, and it remains one of the most widely used non-valved devices^12^. A key limitation, however, is that non-valved designs can overdrain in the early postoperative period, resulting in hypotony (excessively low IOP)^9,13^.

To reduce the risk of postoperative hypotony, the Ahmed glaucoma valve (New World Medical, Inc., California, USA) was introduced in 1993. It is a valved GDD that incorporates pre-tensioned silicone membranes to provide a nominal opening pressure (often reported around ~8–12 mmHg, depending on the model and test conditions)^14^. Although this design reduces the incidence of early overfiltration compared with non-valved devices, hypotony and related complications can still occur in clinical practice^15,16^. In addition, because the resistance of the Ahmed valve is essentially fixed after implantation, some patients may experience insufficient pressure control and persistently elevated IOP (e.g., >21 mmHg), which can compromise long-term outcomes^17^.

To address these limitations, adjustable devices such as the eyeWatch implant (Rheon Medical SA, Lausanne, Switzerland) have emerged. The eyeWatch implant includes a rotatable magnetic disk that applies variable compression on a deformable tube, adjusting fluidic resistance externally via the eyeWatch Pen, a handheld controller^18,19^. Clinical trials showed effective IOP control without hypotony^20^; however, eyeWatch requires manual postoperative adjustment, lacks intrinsic self-adjustability, and raises MRI-compatibility concerns due to its magnetic actuation components. Another magnetically-based device, the “micro-pencil,” introduced by Pereira et al., uses a pencil-tip-shaped actuator to selectively open fluid passageways and reduce IOP^21^. Despite its smaller size and simpler design, the micro-pencil operates only in two distinct resistance states, limiting individualized treatment options. Additionally, like eyeWatch, it requires physician-driven adjustments, and its magnetic components are likely to contribute to higher production costs.

In our previous study, we proposed a self-adjustable glaucoma drainage device (SAGDD) inspired by the Starling resistor principle to autonomously regulate IOP by passively modulating its hydraulic resistance as downstream (bleb) resistance evolves postoperatively^22^. The concept used a thin metallic membrane that remained sealed at low IOP to limit early overdrainage and hypotony, while progressively opening as distal backpressure increased during bleb formation, thereby reducing internal resistance and mitigating late postoperative IOP elevation. Despite promising numerical and in vitro performance, applying and maintaining an external actuation pressure on the membrane within the strict size and integration constraints of an implantable glaucoma device proved impractical. These considerations motivated further design refinement to improve clinical applicability.

In this study, we introduce and evaluate a novel SAGDD, termed eyeValve, designed to overcome key limitations of existing implants, including limited self-adjustability, manufacturing complexity, MRI-compatibility concerns, and device bulk. The central design objective of the eyeValve is to passively regulate IOP within a clinically relevant operating window during the early postoperative period, when downstream (bleb) resistance is still low, and conventional devices are most prone to failure. Specifically, the valve is engineered to maintain IOP above a lower safety threshold of approximately 6 mmHg, consistent with commonly used definitions of numerical hypotony, while simultaneously preventing excessive pressure elevation beyond an upper target of 18 mmHg, a value widely adopted in clinical practice and associated with reduced risk of glaucomatous progression^23,24^. Within this broader physiological window (6–18 mmHg), the eyeValve is designed to operate preferentially in an optimal early postoperative range of 7–13 mmHg, before bleb maturation becomes the dominant determinant of distal outflow resistance.

## Results

### eyeValve design and working principle

**Fig. 1:**
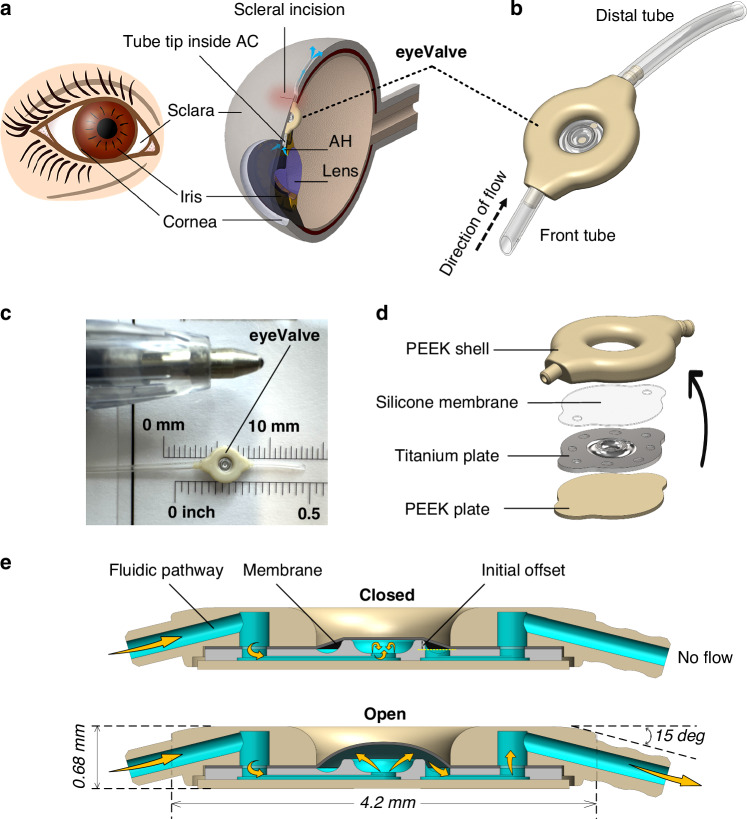
Placement, design, and operating principle of the eyeValve. **a** Eye anatomy indicating the implant location. **b**, **c** Schematic and photograph of the eyeValve connected to inlet and drainage tubes. **d** Exploded view of the valve components. **e** Cross-sectional schematics of the closed and open valve states

The eyeValve is positioned inline between the front tube at the inlet and the distal drainage tube at the outlet (Fig. 1b), forming a compact regulating element within the overall drainage pathway. The device is built as a vertical, sandwich-like stack of four components, top PEEK shell, silicone membrane, titanium plate, and bottom PEEK plate, assembled into a thin, self-contained unit (Fig. 1d). In its fabricated form, the eyeValve remains highly compact (Fig. 1c), measuring 680 µm in thickness and 4.2 mm along its major axis (defined here as the inlet-to-outlet port distance). To better conform to ocular anatomy during implantation, a 15° angle was incorporated between the ports and the valve’s flat surface, facilitating placement along the eye’s curvature while minimizing tissue stress. This compact footprint also enables a simplified implantation concept. Rather than requiring a large scleral flap or an external patch graft, the eyeValve can be inserted into a scleral pocket and covered by scleral tissue (Fig. 1a). In this configuration, the inlet resides in the AC and is exposed to IOP, while the drainage tube tracks along the scleral surface beneath the conjunctiva. AH is diverted from the AC through the front tube, passes through the eyeValve, and exits via the drainage tube, where it is ultimately absorbed by surrounding tissues.

The key functional element enabling the self-adjustable behavior of the eyeValve is a pre-stressed silicone membrane. Pre-stress is established by imposing an initial central offset via the metallic component, which stretches the membrane into a defined initial shape (Fig. 1e). This compliant, pre-loaded membrane dynamically modulates the valve’s hydraulic resistance in response to both proximal and distal pressures, providing fully passive, self-adjustable flow regulation that reduces the risk of early hypotony while mitigating later pressure elevation as distal conditions evolve. Immediately after surgery, before a filtering bleb forms at the drainage site and while distal bleb resistance remains low, the membrane stays nearly sealed against the valve seat, providing sufficient resistance to limit overdrainage (Fig. 1e, closed position). In this regime, if IOP drops below the eyeValve opening threshold, outflow becomes strongly restricted, limiting further pressure reduction. As IOP approaches and exceeds this threshold, the membrane begins to lift, increasing flow and constraining further IOP rise. Over time, as healing progresses and bleb formation increases distal resistance along the AH drainage path, the distal pressure acting on the eyeValve rises accordingly. The resulting pressure beneath the membrane promotes additional lift and enlarges the flow path (Fig. 1e, open position), thereby reducing the valve’s internal resistance to compensate for the increased bleb resistance. In effect, the combined outflow resistance of the valve–bleb system does not increase proportionally with bleb maturation, attenuating IOP elevation and helping maintain IOP within the desired range.

### Numerical parametric analysis and design selection

**Fig. 2:**
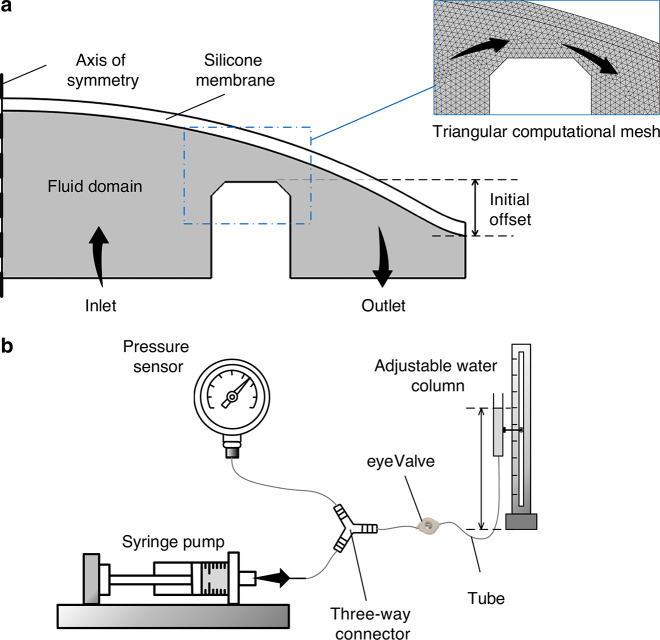
Numerical model and experimental setup of the eyeValve. **a** Schematic of the valve model with applied boundary conditions and a zoomed view of the computational mesh in a critical region. **b** Experimental setup for in vitro testing

To guide fabrication and systematically quantify how key design parameters shape eyeValve performance, fully coupled FSI simulations were conducted in COMSOL Multiphysics (COMSOL AB, Stockholm, Sweden). The model captured the dominant hydraulic restriction at the valve seat by representing the seat–membrane region as a two-dimensional axisymmetric domain (Fig. 2a). The inlet flow was imposed as a velocity boundary condition computed from an assumed physiological AH production rate of 2 µL/min^25^ and the inlet cross-sectional area, while the outlet was prescribed as a pressure boundary that was increased stepwise from 0 to 20 mmHg to emulate progressive distal backpressure associated with bleb formation and maturation. In this framework, bleb development is represented as an increase in distal pressure (i.e., the downstream pressure required to sustain a given AH flow, consistent with Δ*P* = *RQ*), enabling assessment of IOP regulation as downstream outflow conditions become more resistive. The membrane perimeter was fixed to represent clamping within the PEEK housing, and the pre-stressed membrane state was introduced by imposing an initial central offset prior to flow initiation, thereby establishing the intended pre-tension.

**Fig. 3:**
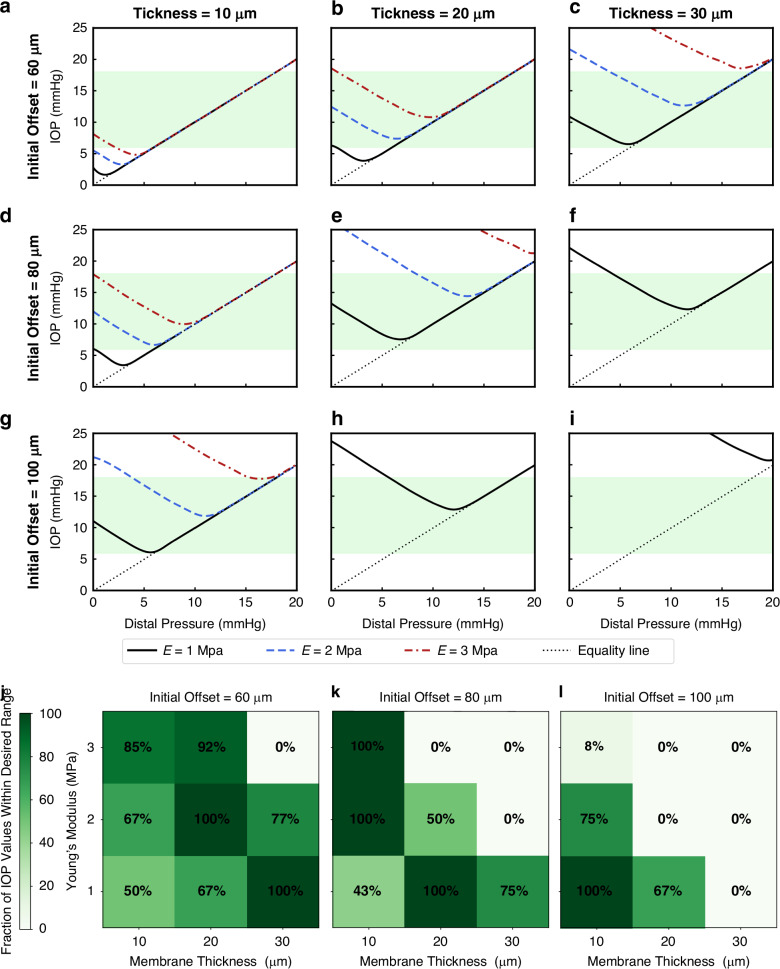
Parametric simulation results for the eyeValve design space. **a**–**i** Simulated pressure characteristics for combinations of membrane thickness (10, 20, and 30 μm; columns from left to right) and initial membrane offset (60, 80, and 100 μm; rows from top to bottom). Within each panel, the pressure characteristics are shown for Young’s moduli of 1 MPa (black solid line), 2 MPa (blue dashed line), and 3 MPa (red dash-dotted line). The dotted line represents the equality line, and the green shaded region indicates the desired IOP range. **j**–**l** Heatmaps showing the fraction of simulated IOP values within the desired pressure range for initial membrane offsets of 60, 80, and 100 μm, respectively, as a function of membrane thickness and Young’s modulus

The top panel of Fig. 3 summarizes the parametric FSI results (27 simulation cases), which were used to map how membrane thickness (10, 20, 30 µm), membrane Young’s modulus (1, 2, 3 MPa), and initial central offset (60, 80, 100 µm) shape pressure regulation, while all other geometric parameters, including the inlet-to-outlet area ratio, were held constant. Here, the initial central offset is the practical design parameter used to generate the prestressed membrane configuration; therefore, its variation also directly quantifies the sensitivity of valve performance to membrane prestress. Across all 27 simulated configurations, the same qualitative regulation behavior was observed. At zero distal pressure, representing the early postoperative condition in which bleb/fibrosis resistance is negligible, the eyeValve imposes a baseline hydraulic resistance to AH outflow that depends on the selected design parameters (i.e., membrane thickness, modulus, and initial offset), leading to higher or lower opening pressures and initial IOP values. As distal pressure is increased (used here as a proxy for rising downstream backpressure during bleb formation), the area-averaged pressure acting beneath the membrane rises, promoting membrane lift, enlarging the flow path, and reducing the valve’s internal resistance. From distal pressures of 0 to approximately 5 mmHg, this reduction in valve resistance consistently outweighed the imposed increase in distal resistance, such that the combined outflow resistance decreased and the predicted IOP transiently dropped compared with the zero–distal-pressure condition. With further distal-pressure elevation, the IOP trends toward the equality line, indicating that the pressure drop across the valve becomes small and the device transitions toward a near-fully open state in which distal resistance increasingly dominates the overall pressure level. Accordingly, when distal pressure exceeded the upper limit of the desired window (green band, 6–18 mmHg), all simulated configurations predicted IOP values above 18 mmHg, reflecting the fact that even a fully open valve cannot compensate for severely elevated distal backpressure.

Within this overall behavior, parameter-dependent shifts in the IOP–distal-pressure curves were clearly observed: increasing membrane thickness, Young’s modulus, or initial offset consistently displaced the curves upward, consistent with higher effective membrane stiffness and higher opening pressures. Stiffer configurations reduced the likelihood of low-pressure hypotony but, in some cases, drove IOP above the desired physiological window, whereas more compliant configurations favored lower IOP but could increase the risk of hypotony. Within the investigated parameter set, six configurations maintained IOP within the predefined physiological window across the full distal-pressure range. These included two configurations at an initial offset of 60 µm (t = 20 µm, E = 2 MPa; and t = 30 µm, E = 1 MPa; Fig. 3b, c), three configurations at an initial offset of 80 µm (t = 10 µm with E = 2 or 3 MPa; and t = 20 µm with E = 1 MPa; Fig. 3d–f), and one configuration at an initial offset of 100 µm (t = 10 µm, E = 1 MPa; Fig. 3g). In contrast, most other parameter combinations produced either sub-physiological pressures (IOP < 6 mmHg) or supra-physiological pressures (IOP > 18 mmHg) over at least part of the distal-pressure range, indicating an increased risk of under- or overdrainage depending on the selected design parameters.

The bottom panel of Fig. 3 presents a quantitative heatmap showing, for each combination of initial offset, membrane thickness, and Young’s modulus, the extent to which IOP was maintained within the predefined physiological window over distal pressures of 0–18 mmHg. Coverage (%) was defined as the fraction of distal-pressure operating points (0–18 mmHg) for which IOP remained within the target window. For the 60 µm initial offset (Fig. 3j), performance was comparatively consistent across the parameter space, with a mean coverage of ~76% across parameter combinations. At a fixed modulus of 1 MPa, increasing membrane thickness progressively improved window coverage from 50% (10 µm; hypotony at approximately half of the operating points) to 67% (20 µm; hypotony reduced to roughly one-third of the operating points), reaching 100% at 30 µm (no hypotony and no excursions above the window). In contrast, for higher stiffness values (2–3 MPa), increasing thickness from 10 µm to 20 µm increased coverage by mitigating hypotony at low distal pressures; however, a further increase to 30 µm shifted the limiting behavior to elevated IOP at higher distal pressures, reducing coverage due to excursions above the target window.

In contrast, the 100 µm offset condition was generally suboptimal. It produced comparatively high valve resistance and yielded IOP values above the predefined physiological window in 8 of the 9 parameter combinations tested. Only one configuration (membrane thickness 10 µm, Young’s modulus 1 MPa) maintained IOP within the window across the full distal-pressure range under this offset condition (Fig. 3g), indicating limited robustness and practical adaptability of the 100 µm design choice.

For the 80 µm offset condition, the IOP remained within the target window over ~57% of the evaluated distal-pressure range on average (Fig. 3k). At a membrane thickness of 20 µm, increasing Young’s modulus from 1 MPa to 2 MPa reduced window coverage from 100% to 50%, and further to 0% at 3 MPa. Similarly, at a fixed modulus of 1 MPa, reducing membrane thickness from 30 µm to 20 µm eliminated the portion of the operating range with IOP above the target window, increasing coverage from 75% to 100%; reducing thickness further to 10 µm shifted the limitation from elevated IOP to hypotony, with 57% of the operating range falling below the hypotony threshold and the remaining 43% within the target window. Among the six configurations achieving full window coverage across the investigated distal-pressure range, three corresponded to the 80 µm offset condition, and the configuration with 80 µm offset, 20 µm thickness, and 1 MPa modulus was selected for fabrication.

### Numerical flow-rate sensitivity analysis

**Fig. 4:**
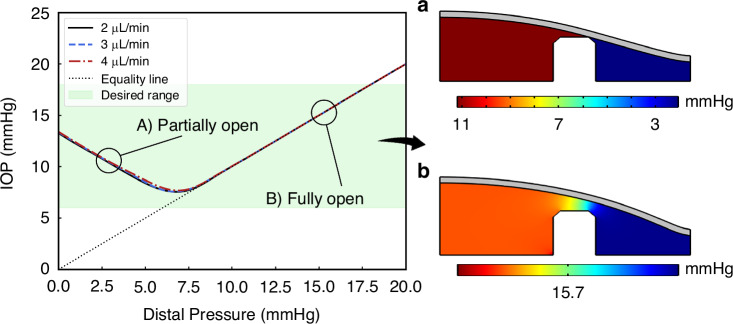
Effect of inlet flow rate on eyeValve pressure characteristics. IOP versus distal pressure at three inlet flow rates. **a**, **b** Pressure distributions within the valve at the partially open (A) and fully open (B) operating points, respectively

After identifying a set of design parameters that satisfied the pressure-regulation criteria in the parametric analysis, a flow-rate sensitivity study was conducted to assess whether the selected eyeValve configuration remains robust to physiological variations in AH production. The analysis focused on the down-selected design (initial offset 80 µm, membrane thickness 20 µm, Young’s modulus 1 MPa) and evaluated its pressure-regulation behavior over inlet flow rates ranging from 2 to 4 µL/min, spanning typical daytime AH production and most of the reported physiological range (≈1.8–4.3 µL/min)^26^. Across all tested flow rates, the IOP–distal-pressure curves nearly overlapped (Fig. 4), indicating minimal sensitivity to changes in inflow. The maximum difference in predicted IOP between flow conditions remained below 0.2 mmHg over the full distal-pressure range, demonstrating that pressure regulation is governed primarily by the pressure-responsive membrane mechanics rather than by flow-rate variations.

For this selected configuration, the valve opening pressure at zero distal pressure was approximately 13 mmHg for all flow rates, well within the desired physiological window (6–18 mmHg). As distal pressure increased, the area-averaged pressure acting beneath the membrane increased, promoting membrane lift and progressively reducing valve resistance. This transition is illustrated by two representative operating points. At point A (distal pressure ≈ 3 mmHg), the valve is partially open, yielding an IOP of approximately 11 mmHg and a pronounced pressure gradient across the valve. At point B (distal pressure ≈ 15.7 mmHg), the membrane is largely lifted, the flow path is fully developed, and the pressure drop across the valve becomes negligible (Pin ≈ Pout).

### In vitro experimental validation

The numerically down-selected eyeValve configuration (initial offset 80 µm, membrane thickness 20 µm, and membrane Young’s modulus 1 MPa) was fabricated and evaluated in vitro using the benchtop flow loop shown in Fig. 2b. At a constant inflow of 2 µL/min, proximal pressure (analogous to IOP) was recorded in real time while distal pressure was increased stepwise from 0 to 20 mmHg; for each distal-pressure condition, the proximal pressure stabilized to a clear plateau, and the steady value was averaged across repeated measurements.

**Fig. 5:**
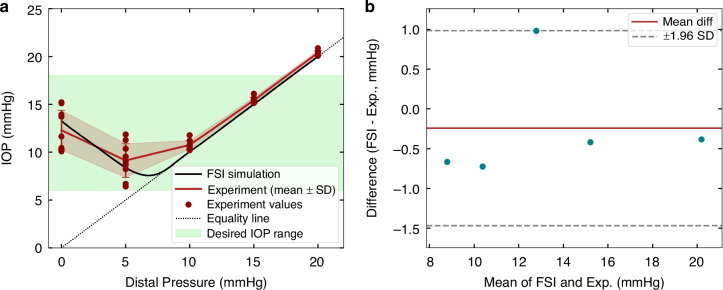
Experimental and numerical assessment of the eyeValve. **a** Experimental and simulated IOP data. **b** Bland–Altman comparison

Figure 5a compares the measured pressure regulation of nine fabricated prototypes with the corresponding FSI predictions over distal pressures of 0, 5, 10, 15, and 20 mmHg. The experiments reproduced the same overall regulation trend observed numerically. At zero distal pressure, the measured IOP was 12.3 ± 2.0 mmHg, closely matching the simulated value (13.3 mmHg). When distal pressure was increased to 5 mmHg, IOP decreased to 9.1 ± 1.7 mmHg experimentally (8.4 mmHg in simulation), consistent with the transition to partial opening and a net reduction in valve resistance. With further increases in distal pressure, the valve approached a near-fully open state and contributed only a small additional pressure drop; for example, at 10 mmHg distal pressure, the inlet and outlet pressures became similar and IOP remained close to the distal pressure (10.7 ± 0.4 mmHg experimentally; 10.0 mmHg in simulation).

Agreement between simulations and experiments is further quantified in Fig. 5b using a Bland–Altman analysis performed on the experimental mean IOP at each distal-pressure condition (*n* = 5 paired conditions; mean computed across the nine valves). The mean bias (FSI − Exp.) was −0.24 mmHg, indicating a slight overall underprediction by the model. The 95% limits of agreement were −1.47 to +0.98 mmHg (SD = 0.62 mmHg), and all paired points fell within these limits. Together, these results demonstrate close correspondence between the FSI predictions and in vitro measurements across the investigated operating range, with minimal systematic bias.

### Ex vivo surgical feasibility assessment

**Fig. 6:**
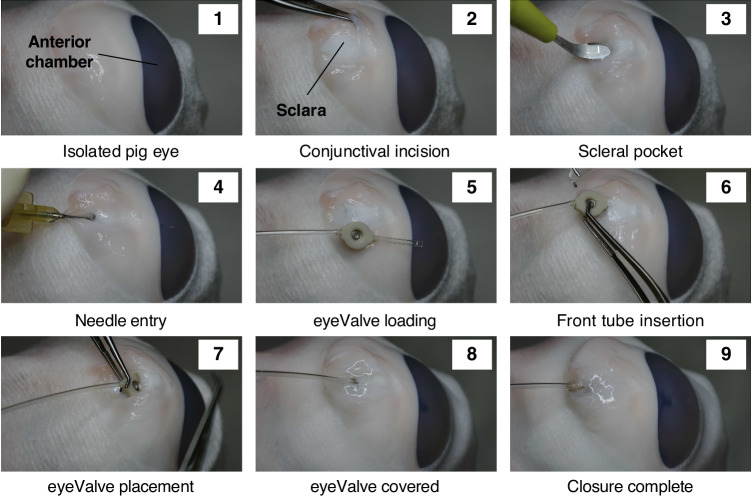
Surgical implantation sequence of the eyeValve in an enucleated pig eye. Sequential steps showing conjunctival peritomy, scleral pocket formation, AC access, valve insertion, and closure

In order to assess surgical feasibility and anatomical compatibility, ex vivo implantation was performed in enucleated porcine eyes. The results demonstrate that the eyeValve can be implanted using a standard surgical workflow and that its compact form factor enables stable positioning beneath the sclera. The sequential implantation steps are shown in Fig. 6. In brief, a scleral pocket was created, the inlet tube was introduced into the AC through a controlled scleral entry, and the valve body was seated beneath the sclera prior to closure. Across all procedures, implantation was completed without technical difficulty using conventional microsurgical instruments. Following placement, the device exhibited a secure anatomical fit within the porcine eye. The valve body sat flush against the sclera within the prepared pocket, and the inlet tube remained appropriately positioned within the AC. Its intraocular placement follows the same general principle as conventional GDD tubes and is therefore not expected to interfere with normal iris function, provided that appropriate surgical positioning is maintained. The observations support the procedural feasibility of scleral-pocket implantation and confirm that the eyeValve geometry is compatible with ocular anatomy in a clinically relevant ex vivo model.

## Discussion

### Robustness, novelty, and performance of the eyeValve

In this study, we developed and evaluated a miniature SAGDD (eyeValve) designed to provide autonomous IOP regulation without external intervention. The design goal was to maintain IOP within the physiological window (6–18 mmHg) while targeting an opening pressure in the optimal band of 7–13 mmHg, corresponding to the early postoperative period when distal (bleb) resistance is still low, and overfiltration is the primary concern. In parallel, the proposed architecture was conceived to address practical limitations of existing GDDs, including limited self-adjustability, manufacturing complexity, MRI-compatibility concerns, and device footprint.

Unlike commercially available fixed-resistance implants, such as the widely used Ahmed glaucoma valve, eyeValve modulates its hydraulic resistance through a passive, intrinsic mechanism. As distal pressure rises, used here as a proxy for increasing downstream backpressure associated with bleb formation and fibrotic encapsulation, the valve progressively opens and reduces its internal pressure drop, thereby attenuating the transmission of distal-pressure increases to the inlet. This behavior contrasts with fixed-resistance devices, for which the device resistance remains approximately constant; consequently, increases in distal resistance/backpressure tend to translate more directly into elevated IOP. Clinically, insufficient pressure control in such settings can motivate additional postoperative interventions (e.g., needling, revisions) and is associated with suboptimal long-term outcomes reported for some fixed-resistance GDDs^27^.

**Fig. 7:**
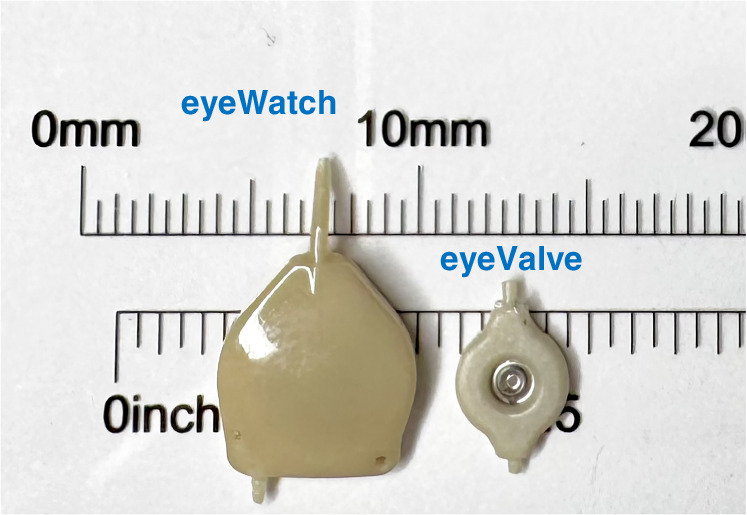
Size comparison of eyeValve and eyeWatch

In addition to fixed-resistance valves, eyeValve also mitigates several practical limitations associated with magnetically adjustable systems such as eyeWatch. Adjustable devices like eyeWatch represent an important clinical advance by enabling postoperative titration to patient-specific needs, which can be particularly valuable during the dynamic early healing phase. However, this benefit comes at the cost of follow-up dependence and added device complexity. In contrast, eyeValve relies on a fully passive, self-adjustable architecture that does not require postoperative external adjustments, thereby simplifying follow-up and reducing dependence on clinician-driven titration. The absence of magnetic components and mechanically complex actuation mechanisms further reduces system complexity and alleviates MRI-compatibility concerns, while also avoiding the device bulk associated with permanent magnets. Importantly, the compact form factor of eyeValve supports straightforward implantation and enables complete coverage by native scleral and conjunctival tissues without a patch graft. This practical advantage was supported by the ex vivo porcine-eye feasibility procedures, in which the device could be seated securely within a scleral pocket using standard microsurgical steps and achieved an appropriate anatomical fit. Avoiding a patch graft can reduce operative steps and foreign material burden, which may lower the risk of graft-related inflammation and late conjunctival erosion while simplifying the surgical workflow. This size advantage relative to eyeWatch is illustrated in Fig. 7, which qualitatively compares device footprints and highlights the substantially smaller profile of eyeValve.

Beyond its self-adjusting function, eyeValve exhibited minimal sensitivity to variations in flow rate. Across physiologically relevant AH production rates (2–4 µL/min), the regulated IOP changed only marginally, which is clinically relevant because AH production differs between patients and can fluctuate over time (e.g., diurnal variation and medication effects). Limited flow dependence therefore supports stable pressure control without requiring patient-specific tuning of the design or operating setpoint. In addition to these functional advantages, eyeValve offers flexibility in surgical implementation. The valve mechanism can be incorporated into a conventional tube–plate configuration, consistent with established GDD practice, or deployed in a more compact, standalone manner using an extended drainage tube without a dedicated plate. The tube–plate approach may be preferable in patients who require a larger subconjunctival surface area to accommodate a strong fibrotic response, whereas a plate-free configuration could reduce implant footprint, tissue disruption, and surgical complexity. The long-term performance of a standalone strategy, as well as the possible influence of posture- and motion-related effects, remains to be established, although the latter are expected to be limited because the eyeValve operates over a short local drainage path rather than across a large hydrostatic pressure column. Comparative studies will nevertheless be needed to define the optimal indications for each configuration across different patient profiles.

Taken together, these results highlight eyeValve’s distinct advantages: autonomous self-adjustability, robustness to physiological variation in AH production, straightforward implantation enabled by its compact form factor, and potential cost-effectiveness arising from a simplified architecture and fabrication workflow. Importantly, the close agreement between the FSI predictions and the in vitro measurements across distal-pressure conditions supports the robustness of the underlying regulation mechanism and the validity of the reduced modeling framework. The small bias and narrow limits of agreement indicate that the model captures the dominant seat–membrane behavior and can be used to guide parameter selection while reducing reliance on iterative prototyping. These features position eyeValve as a promising candidate for both primary surgical intervention and the management of refractory glaucoma, potentially addressing key gaps that persist with current commercially available GDDs.

### Translational challenges and future directions

The present study evaluated the eyeValve from several key engineering and early translational perspectives, including its pressure-regulating concept, miniaturized architecture, and surgical feasibility. Under controlled benchtop conditions, the core operating principle of the device was established, while ex vivo porcine-eye experiments were used specifically to assess surgical handling and anatomical compatibility. These findings provide an initial foundation for translation, but several important questions remain regarding device behavior after implantation.

An important translational challenge concerns the biological environment surrounding the implant. Upon implantation, the eyeValve will interface with living tissues rather than a controlled atmosphere. This introduces the possibility of external tissue loading and fibrotic encapsulation, which could subject the membrane to mechanical loads not present in benchtop testing. In addition, biological fouling, arising from protein deposition within the internal fluid path or tissue ingrowth, could interfere with membrane mobility or alter hydraulic resistance over time. The ex vivo models used here were not intended to provide a definitive assessment of postoperative valve function in a glaucomatous setting. Because these experiments were performed in normal enucleated eyes, they could not fully reproduce the sealed living ocular and periocular environment, including realistic tissue support, external loading, and disease-relevant pressure conditions.

Despite these potential challenges, prior literature on membrane-based drainage concepts suggests that this general regulating mechanism may remain feasible under biological conditions^28^. However, this must still be confirmed for the specific eyeValve architecture through dedicated animal experiments. Such studies will be essential to determine the long-term stability of the opening behavior, the durability of the membrane, and the sustained biocompatibility of the device materials in vivo.

If biological factors are found to significantly influence performance, several design strategies remain available. To mitigate external tissue pressure and fibrotic interference, future iterations could incorporate protective structural features, such as a rigid housing or shroud, to shield the membrane while preserving the local fluid space required for regulation. In addition, if in vivo data identify a consistent performance shift due to biological loading, the device characteristics could be further tuned during manufacturing through adjustment of parameters such as the initial membrane offset, membrane thickness, or material properties.

Beyond the biological interface, further technical refinement will also be required for clinical translation. While prototype-to-prototype agreement in this study was acceptable, inter-device variability remains partly influenced by membrane tolerances and assembly repeatability. Tighter control of membrane thickness and a more standardized assembly process are expected to improve batch consistency. In parallel, practical aspects such as sterilization compatibility, shelf-life, and long-term mechanical durability will need to be established. Together, these steps will be important for advancing the eyeValve from a validated mechanical concept toward a reliable medical-grade technology for glaucoma management.

## Materials and methods

### Numerical simulations

#### FSI model setup, boundary conditions, and solver settings

FSI simulations were performed in COMSOL Multiphysics (COMSOL AB, Stockholm, Sweden) using a two-dimensional axisymmetric model of the valve seat–membrane region (Fig. 2a). Non-axisymmetric upstream and downstream features were not included; the model therefore represents an axisymmetric “equivalent seat” approximation intended to capture the dominant seat-driven opening and resistance behavior. At the inlet, flow was imposed as an axial velocity boundary condition, with inlet velocity computed from an assumed AH production rate of 2 µL/min^25^ and the inlet cross-sectional area. At the outlet, a pressure boundary condition was applied and ramped from 0 to 20 mmHg. The membrane perimeter was fixed to represent clamping within the PEEK housing.

Membrane prestress was introduced numerically through a prescribed moving-mesh displacement applied to the valve-seat region prior to flow analysis. Specifically, the seat boundaries were displaced upward in the axial direction until the prescribed initial offset was reached, thereby deforming the membrane into its preloaded configuration. This displacement was imposed using a smooth ramp function to avoid abrupt numerical transients. Once the target offset was achieved, the deformed configuration was maintained, and the subsequent FSI response was computed from this prestressed state. Accordingly, membrane prestress was not prescribed as an independent stress value, but generated geometrically through the imposed initial offset. This approach closely reflects the physical device, in which the membrane is prestressed by the assembled geometry and the imposed seat offset rather than by an externally applied pre-tension.

Simulations were solved using a time-dependent, fully coupled approach. Nonlinear iterations were handled using a Newton–Raphson scheme, and time integration used the Backward Differentiation Formula (BDF). Convergence was monitored based on the relative change in the global solution vector (fluid pressure and velocity, solid displacement, and mesh deformation). Simulations were considered converged when the normalized solution update fell below 0.01; this tolerance was validated by comparison with a stricter criterion of 0.001, yielding differences of less than 0.07%.

#### Material properties

The working fluid was modeled as pure water with a density of 998 kg/m³ and a dynamic viscosity of 1.002 × 10⁻³ Pa·s to represent standard properties of AH^29^. The silicone membrane, which constitutes the core functional element of the valve, was modeled as a linear elastic material. This assumption is supported by the observation that average deformations during physiological operation remained predominantly within the linear strain regime (strains less than 10%), where the material exhibits a nearly linear stress–strain relationship^30^. The elastic modulus of the membrane was determined using established empirical correlations between Shore A hardness and Young’s modulus for elastomers, as originally described by Gent^31^ and validated in subsequent studies^32^. Accordingly, three Young’s modulus values, 1, 2, and 3 MPa, corresponding approximately to silicone membranes with Shore A hardness values of 30, 40, and 50, were selected for simulation.

A Poisson’s ratio of 0.49 was assigned to the membrane, reflecting the near-incompressibility of silicone elastomers and aligning with values commonly reported in the literature^33^, and the silicone density was set to 1100 kg/m³, consistent with reported densities for silicone rubbers^34,35^. Because results were extracted after the response stabilized at each ramped distal-pressure level, inertial effects are unlikely to influence the reported IOP–distal-pressure relationship, and the precise density value is not expected to significantly affect the conclusions. Table 1 summarizes the material details used in the numerical simulations.

**Table 1:** Material properties used in the numerical simulations

Property	Value	Notes/Source
Fluid density	998 kg/m³	Water at 293 K
Fluid viscosity	1.002 × 10⁻³ Pa·s	Water at 293 K
Fluid behavior	Newtonian	–
Young’s modulus (E)	1, 2, 3 MPa	30, 40, 50 Shore A^32^
Poisson’s ratio	0.49	Near-incompressible assumption^33^
Silicone density	1100 kg/m³	Typical for medical-grade silicones
Material model	Linear elastic	Valid for strains <10%

#### Mesh generation and adaptive remeshing

A high-quality triangular mesh was generated across the entire computational domain, with the maximum element size set to 0.00675 mm to ensure sufficient spatial resolution. A magnified view of the mesh in the critical region of interest is presented in Fig. 2a. Mesh quality was assessed in COMSOL using the skewness-based element quality metric (Quality measure: *Skewness*). The mesh achieved a minimum element quality greater than 0.5 and an average element quality greater than 0.85 (values reported by COMSOL on a 0–1 scale, where higher values indicate better element shape). To ensure that the simulation results were independent of mesh resolution, a mesh convergence study was performed by progressively refining the mesh and monitoring the valve inlet pressure as the key output variable. Mesh independence was confirmed when additional refinement produced less than a 2% change in the predicted valve opening pressure (the inlet pressure at 0 distal pressure). Additionally, an automatic remeshing algorithm was employed to prevent a loss of mesh quality due to membrane deformation during simulation. Automatic remeshing was triggered whenever the element quality (skewness measure) dropped below 0.3, maintaining mesh quality throughout the simulation.

### Valve fabrication and experimental characterization

#### Valve fabrication and in vitro testing

Titanium plate and the PEEK components were fabricated using a high-precision, 5-axis micro-computer numerical control (CNC) milling machine (DATRON C5). CNC toolpaths were generated with Mastercam software (CNC Software, Inc., Tolland, CT, USA), based on detailed 3D models constructed in SolidWorks (Dassault Systèmes, Vélizy-Villacoublay, France). The membrane was cut to the desired size using a custom-made metallic punch. Following fabrication, each component, including the membrane, underwent ultrasonic cleaning and microscopic inspection to ensure the complete removal of manufacturing residues and surface contaminants.

Assembly was performed in several controlled steps using a dedicated assembly setup. First, the titanium plate was bonded to the PEEK plate with epoxy resin, forming the lower subassembly from the two bottom components shown in Fig. 1d. Simultaneously, the silicone membrane was positioned against the inner flat surface of the top PEEK shell. During this step, isopropanol was used as a temporary lubricant to facilitate precise centering; upon evaporation, the membrane’s natural surface tack ensured uniform, unstressed adhesion to the PEEK surface.

This temporary fixation allowed the initial central offset, and thus the membrane prestress, to be applied in a controlled geometric manner during final assembly. Before joining the two subassemblies, epoxy resin was applied along the inner bonding ledge of the top PEEK shell, and a small amount of silicone adhesive was deposited with a fine-tip applicator into the six grooves of the titanium plate to further secure the membrane and prevent displacement after closure. The upper and lower subassemblies were then brought together and bonded under controlled compression using a precision alignment guide, while a controlled load was applied during curing at room temperature to maintain the intended sandwich configuration. This standardized “sandwich” compression ensured a consistent mechanical seal and a reproducible initial membrane configuration across all prototypes.

In vitro characterization was performed using the benchtop flow loop shown in Fig. 2b. Water was delivered through the valve using a syringe pump at a constant flow rate of 2 µL/min. Proximal pressure was measured in real time using a manometer connected to a data acquisition board. Distal pressure was applied using an adjustable water column connected to the outlet and varied from 0 to 20 mmHg. At each distal-pressure level, proximal pressure was recorded after reaching a stable plateau; measurements were repeated multiple times per condition and averaged to obtain the reported values.

#### Ex vivo surgical implantation in porcine eye model

Ex vivo implantation was performed in enucleated porcine eyes to evaluate procedural feasibility and device fit. Porcine eyes were selected due to their close anatomical similarity to human eyes, including comparable globe size, scleral thickness, and AC geometry^36,37^. Prior to implantation, each eye was stabilized on a custom mounting platform to enable controlled manipulation during microsurgery. The procedure began with conjunctival incision, followed by creation of a 4.5 × 4.5 mm scleral pocket using a crescent sapphire blade. The pocket was dissected to a depth extending to approximately 1 mm from the limbus to define the intended implantation site. A 24-gauge needle was then used to create a controlled scleral entry into the AC. The inlet tube was advanced through this entry and positioned within the AC, with care taken to avoid contact with the cornea and iris. The valve body was placed within the scleral pocket, after which the scleral pocket and conjunctival incision were closed to secure the implant and restore ocular surface integrity.

## Data Availability

The data that support the findings of this study, including numerical simulation results and in vitro and ex vivo experimental measurements, are available from the corresponding author upon reasonable request.
